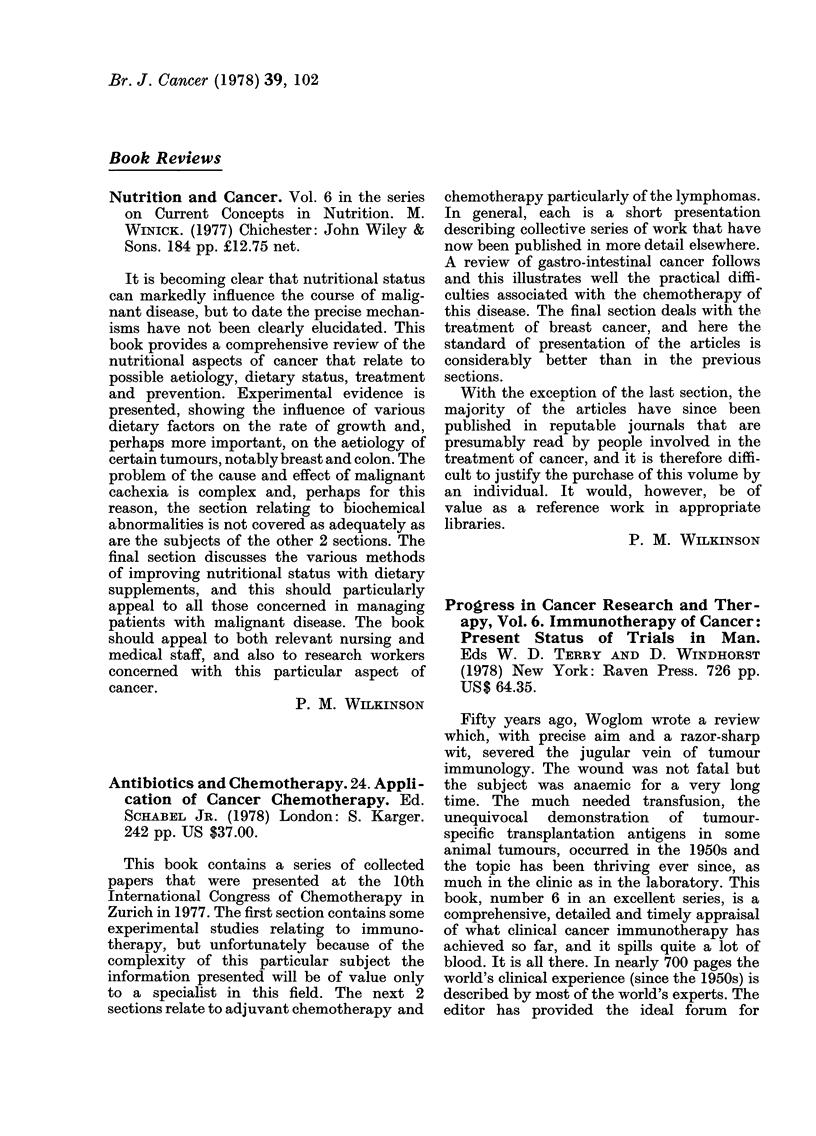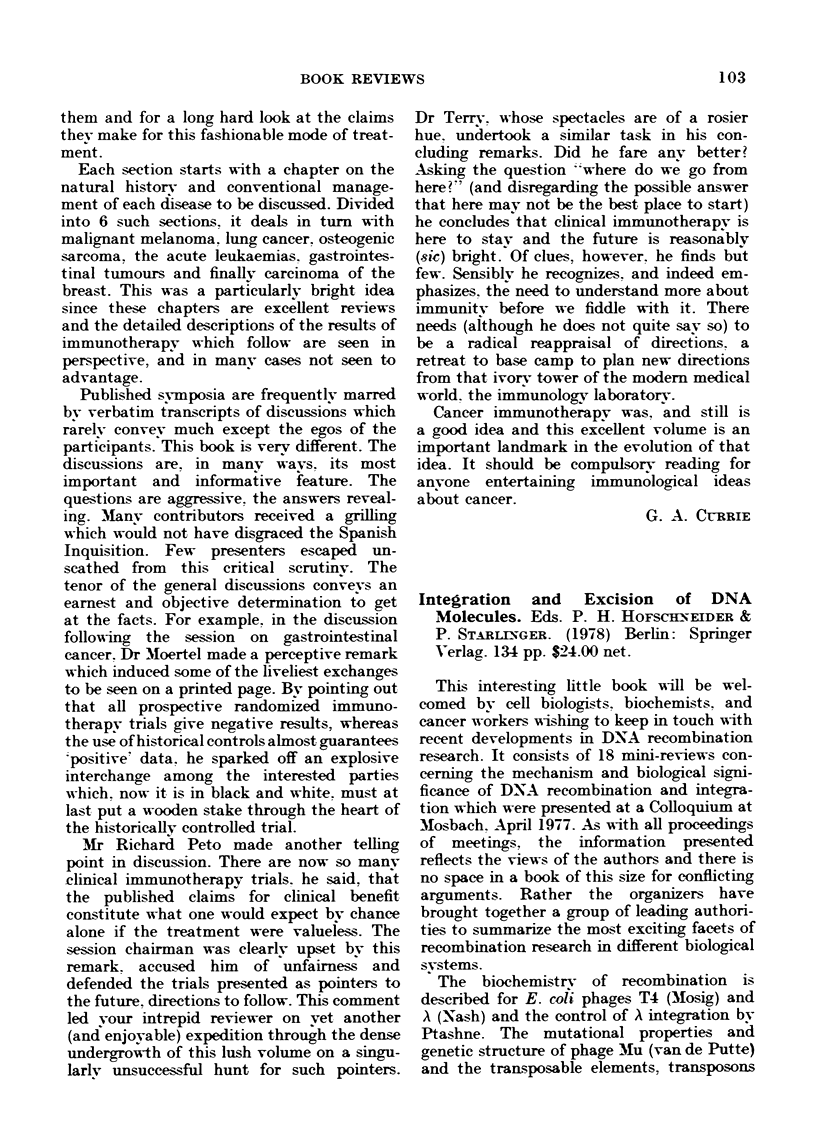# Progress in Cancer Research and Therapy, Vol.6. Immunotherapy of Cancer: Present Status of Trials in Man

**Published:** 1979-01

**Authors:** G. A. Currie


**Progress in Cancer Research and Therapy, Vol. 6. Immunotherapy of Cancer: Present Status of Trials in Man.** Eds W. D. Terry and D. Windhorst (1978) New York: Raven Press. 726 pp. US$ 64.35.

Fifty years ago, Woglom wrote a review which, with precise aim and a razor-sharp wit, severed the jugular vein of tumour immunology. The wound was not fatal but the subject was anaemic for a very long time. The much needed transfusion, the unequivocal demonstration of tumour-specific transplantation antigens in some animal tumours, occurred in the 1950s and the topic has been thriving ever since, as much in the clinic as in the laboratory. This book, number 6 in an excellent series, is a comprehensive, detailed and timely appraisal of what clinical cancer immunotherapy has achieved so far, and it spills quite a lot of blood. It is all there. In nearly 700 pages the world's clinical experience (since the 1950s) is described by most of the world's experts. The editor has provided the ideal forum for them and for a long hard look at the claims they make for this fashionable mode of treatment.

Each section starts with a chapter on the natural history and conventional management of each disease to be discussed. Divided into 6 such sections, it deals in turn with malignant melanoma, lung cancer, osteogenic sarcoma, the acute leukaemias, gastrointestinal tumours and finally carcinoma of the breast. This was a particularly bright idea since these chapters are excellent reviews and the detailed descriptions of the results of immunotherapy which follow are seen in perspective, and in many cases not seen to advantage.

Published symposia are frequently marred by verbatim transcripts of discussions which rarely convey much except the egos of the participants. This book is very different. The discussions are, in many ways, its most important and informative feature. The questions are aggressive, the answers revealing. Many contributors received a grilling which would not have disgraced the Spanish Inquisition. Few presenters escaped unscathed from this critical scrutiny. The tenor of the general discussions conveys an earnest and objective determination to get at the facts. For example, in the discussion following the session on gastrointestinal cancer, Dr Moertel made a perceptive remark which induced some of the liveliest exchanges to be seen on a printed page. By pointing out that all prospective randomized immunotherapy trials give negative results, whereas the use of historical controls almost guarantees 'positive' data, he sparked off an explosive interchange among the interested parties which, now it is in black and white, must at last put a wooden stake through the heart of the historically controlled trial.

Mr Richard Peto made another telling point in discussion. There are now so many clinical immunotherapy trials, he said, that the published claims for clinical benefit constitute what one would expect by chance alone if the treatment were valueless. The session chairman was clearly upset by this remark, accused him of unfairness and defended the trials presented as pointers to the future, directions to follow. This comment led your intrepid reviewer on yet another (and enjoyable) expedition through the dense undergrowth of this lush volume on a singularly unsuccessful hunt for such pointers. Dr Terry, whose spectacles are of a rosier hue, undertook a similar task in his concluding remarks. Did he fare any better? Asking the question "where do we go from here?" (and disregarding the possible answer that here may not be the best place to start) he concludes that clinical immunotherapy is here to stay and the future is reasonably (*sic*) bright. Of clues, however, he finds but few. Sensibly he recognizes, and indeed emphasizes, the need to understand more about immunity before we fiddle with it. There needs (although he does not quite say so) to be a radical reappraisal of directions, a retreat to base camp to plan new directions from that ivory tower of the modern medical world, the immunology laboratory.

Cancer immunotherapy was, and still is a good idea and this excellent volume is an important landmark in the evolution of that idea. It should be compulsory reading for anyone entertaining immunological ideas about cancer.

G. A. CURRIE